# Circular RNA hsa_circ_0000144 aggravates ovarian Cancer progression by regulating ELK3 via sponging miR-610

**DOI:** 10.1186/s13048-022-01048-3

**Published:** 2022-10-15

**Authors:** Dandan Wu, Jia Liu, Liji Yu, Shaofang Wu, Xiaomei Qiu

**Affiliations:** Department of Obstetrics and Gynecology, The First Hospital of Quanzhou Affiliated to Fujian Medical University, Quanzhou, 362000 China

**Keywords:** circ_0000144, miR-610, ELK3, Ovarian cancer, Proliferation

## Abstract

**Background:**

Ovarian cancer is a common cause of death among women and a health problem worldwide. Circ_0000144 has been confirmed to be an oncogene involved in cancer progression, such as gastric cancer. However, the role of circ_0000144 in ovarian cancer remains unclear and needs to be elucidated. This retrospective study aimed to investigate the underlying mechanism of circ_0000144 in ovarian cancer.

**Methods:**

Differentially expressed circ_0000144 expression in ovarian cancer and normal tissues was identified by quantitative reverse transcriptase polymerase chain reaction (qRT-PCR). In vitro assays were performed to explore the biological functions of circ_0000144 in ovarian cancer cells. An in vivo xenograft model was used to investigate the efficacy of circ_0000144 in the progression of ovarian cancer.

**Results:**

Circ_0000144 was significantly upregulated in ovarian cancer cells and tissues. Circ_0000144 overexpression significantly promoted ovarian cancer cell proliferation, migration, and invasion. This study further demonstrated that circ_0000144 downregulated ELK3 levels by sponging miR-610 in ovarian cancer cells. Moreover, circ_0000144 significantly promotes ovarian cancer tumorigenesis in vivo.

**Conclusion:**

Our data indicate that circ_0000144 could enhance the carcinogenesis of ovarian cancer by specifically targeting miR-610, which may serve as a novel target for the diagnosis and prognosis of ovarian cancer.

## Introduction

Despite great progress in the diagnosis and treatment of cancer over the past decade, ovarian cancer is one of the three most common gynecological malignancies and has the highest mortality rate among gynecological tumors worldwide [1]. Despite continuous progress in surgical, chemotherapy, and radiotherapy treatments, the prognosis of ovarian cancer remains unsatisfactory, and its morbidity and mortality are increasing annually [2, 3]. Therefore, there is an urgent need to identify potential biomarkers for early diagnosis to obtain optimal clinical antitumor therapies.

In eukaryotes, circular RNAs (circRNAs) are produced by reverse splicing of mRNA precursors of exons of thousands of genes [4]. CircRNAs are highly abundant and specifically expressed in tissues, and thousands of circRNAs are expressed differently in tumor and normal tissues [5]. Previous studies have shown that circRNAs regulate gene expression via multiple mechanisms, such as sponging of microRNAs (miRNAs) [6]. CircRNAs are involved in many biological functions, especially in cell cycle regulation and extracellular interactions, and play gene regulatory roles in multicellular organisms through interactions with nucleic acids, proteins, and especially microRNAs [7]. The regulation of circRNAs on gene expression controls a variety of biological functions, such as cell growth and apoptosis, development, embryogenesis and the pathogenesis of human diseases, especially cancer. Dysregulated circRNAs play a role in biological processes including cell proliferation, migration, invasion, apoptosis and angiogenesis, thereby affecting tumorigenesis [8, 9].

Some circRNAs have been shown to play an important role in ovarian cancer progression and have been used as biomarkers for the diagnosis and prognosis of ovarian cancer. For instance, circ_0078607 contributes to ovarian cancer carcinogenesis by sponging oncogenic miR-518a-5p to induce FAS expression [10]. However, the development of cancer is complex and may involve multiple signaling pathways. Therefore, the expression profiles and functions of circRNAs in human ovarian cancer need to be investigated. Recently, circ_0000144 was discovered to be an important factor in various tumors [11, 12]. Specifically, circ_0000144 overexpression has been found in gastric cancer (GC) [13] and can also promote the development of bladder cancer by regulating miRNAs [14]. However, its biological function in ovarian cancer remains unknown.

ETS transcription factor ELK3 (ELK3) is an ETS domain protein that forms a ternary complex with DNA and serum response factor (SRF) [15]. ELK3 is a transcription suppressor that is converted into a transcription activator by phosphorylation of extracellular signal-regulated kinase 1/2 (ERK1/2) in response to Ras signaling [16]. ELK3 is highly expressed in a variety of cancers, including basal-like malignant breast cancer, and coordinates metastasis during tumor progression [17]. Previous studies have shown that ELK3 plays a vital role in the development of breast and bladder cancer [18]. Overexpression of ELK3 also occurs in ovarian cancer cell lines and human tumors [19]. However, the potential gene regulatory mechanism of ELK3 in human ovarian cancer remains unclear. Therefore, we investigated the association between circ_0000144 and ELK3 while detecting the expression of circ_0000144 in ovarian cancer.

In the present study, we first identified that circ_0000144 is significantly upregulated in ovarian cancer tissues and cell lines. Functional experiments revealed that circ_0000144 enhanced ovarian cancer cell proliferation, invasion, and migration and promoted tumor growth in mice. Furthermore, mechanistic investigations indicated that circ_0000144 exhibited a tumor promoter role by sponging miR-610 and increasing ELK3 expression in ovarian cancer cells. Moreover, circ_0000144 knockdown significantly inhibit the expression of ELK3 and suppress ovarian cancer progression. This study revealed a probable pathway mechanism of circ_0000144 in ovarian cancer progression.

## Materials and methods

### Samples and cell lines

Primary ovarian cancer samples were obtained from 60 patients diagnosed with ovarian cancer before treatment between August 1, 2013, and June 30, 2016. Tumor and paired non-carcinoma tissue samples were acquired from patients with ovarian cancer at Department of Obstetrics and Gynecology in the First Hospital of Quanzhou Affiliated to Fujian Medical University. Peripheral blood samples were collected from the patients and healthy control subjects when they attended the clinic at the start of the study. Serum was prepared and stored at − 80 °C until further processing. The study protocol was approved by the Medical Ethics Committee of First Hospital of Quanzhou Affiliated to Fujian Medical University and the code of ethical number was 2020–185. The procedures involving human participants in this study were conducted in accordance with the guidelines of the Declaration of Helsinki. Each participant provided informed consent. Human ovarian cancer cell lines (SKOV3, ES-2, and OVCAR3) and normal human ovarian cells (IOSE80) were purchased from the Cell Bank of the Chinese Academy of Sciences (Shanghai, China). All cells were cultured in DMEM (Gibco, USA) containing 10% fetal bovine serum (FBS, Gibco, USA) and 0.1% penicillin-streptomycin and incubated in an incubator at 37 °C and 5% CO_2_.

### Cell transfection

The synthetic circ_0000144 sequence was subcloned into the pcDNA3.1 vector as pcDNA3.1-circ_0000144 vector. Small interfering RNAs (siRNAs) against circ_0000144 (si-circ_0000144, 5′-AGGGAGAGAGAGGTAGAACTA-3′) were used to reduce circ_0000144 expression [13]. MiR-610 mimics were used to overexpress miR-610 in the cells, and NC mimics served as negative controls. MiR-610 inhibitors were used to knock down miR-610 expression in cells. Full-length ELK3 was constructed into pcDNA3.1 vector, and blank pcDNA3.1 was used as a control. Cells were transfected using Lipofectamine 3000 (Invitrogen) according to the manufacturer’s instructions.

### Quantitative reverse transcriptase polymerase chain reaction (qRT-PCR)

Total RNA from tissues and indicated treated cells was extracted with TRIzol reagent. cDNA was synthesized from RNA by reverse transcription using a PrimeScript RT reagent kit (RR036A, Takara Biotechnology, Shiga, Japan). Real-time qPCR was performed in accordance with the SYBR® Premix Ex TaqTM II kit instructions (RR820A, Takara Biotechnology, Shiga, Japan) using the ABI7500 Real-Time Fluorescence Quantitative PCR System (7500, ABI, USA). The relative expression of circ_0000144, miR-610p, and ELK3 was calculated using the 2 ^−ΔΔCt^ method, with glyceraldehyde-3-phosphate dehydrogenase (GAPDH) serving as the endogenous control. Primers were performed the amplification according to previous reports [13, 20], and the sequences were as follows: GAPDH, forward: 5′-TATGATGATATCAAGAGGGTAGT-3′ and reverse: 5′-TGTATCCAAACTCATTGTCATAC-3′; Circ_0000144, forward: 5′-GAGTGTTGGCCTGTCCTCAA-3 and reverse: 5′-TTGTGCCCAGTTGCCTGTAT-3′; SLAMF6, forward, 5′-GAGTGTTGGCCTGTCCTCAA-3′ and reverse, 5′-TTGTGCCCAGTTGCCTGTAT-3′; MiR-610, forward: 5′-GAGCTAAATGTGTGCTGG-3′ and reverse: 5′-GAACATGTCTGCGTATCTC-3′.

### Validation of the sequencing data

The total genomic DNA (gDNA) was extracted using the Blood/Cell/Tissue Genomic DNA Extraction Kit (Tiangen, China) with the manufacturer’s instructions. Based on NCBI reference sequences, convergent and divergent primers were designed using Primer 5.0 software to validate the existence of circRNA. All primers used in this study were synthesized by Sangon (Sangon Biotech, Shanghai, China). For each PCR amplification, cDNA or gDNA was used with 2 × Taq Master Mix (Vazyme, China), and 40 cycles of PCR cycling condition were performed. PCR products were examined by 1% agarose gel electrophoresis. PCR products from that amplified with divergent primers only from cDNA template were sent for Sanger sequencing by Sangon Biotech Co., Ltd.

### EdU assay

SKOV3 and ES-23 cells at a density of 5 × 10^4^ were seeded into a 96-well plate for 24 h, followed by incubation with 50 μmol/l EdU for 2 h at 37 °C. After fixation and permeabilization, the cells were incubated in DAPI solution and observed by fluorescence microscopy (Olympus, Tokyo, Japan).

### RNase R treatment

To examine the stability of hsa_circ_0000144 in SKOV3 and ES-23 cells, 10 μg of total RNA was incubated with RNase R (40 U; Epicenter Biotechnologies, Madison, WI, USA). At 1 h post-digestion, the enrichment of hsa_circ_0000144 and GAPDH was analyzed using RT-qPCR.

### Transwell invasion

The SKOV3 and ES-23 cells were transfected with the indicated plasmids. The medium in the lower chamber contained 10% FBS, and the cells (5 × 105) suspended in Matrigel were added to the upper chambers at the same time. The cells were incubated at 37 °C for 72 h. Cells that passed through the membrane were stained with methanol and 0.1% crystal violet and photographed. Cell invasion was quantified using direct microscopic visualization and counting.

### Luciferase reporter assay

Plasmid constructs carrying wild-type or mutant circ_0000144/ELK3 3UTR in the psiCHECK vector were co-transfected with miR-610 mimic or miR-NC into SKOV3 and ES-23 cells, respectively, using Lipofectamine 2000 (Thermo Fisher Scientific, USA). The cells were lysed 48 h after transfection, and luciferase activity was determined using the dual-luciferase reporter assay method. The PierceTM Renilla-Firefly Luciferase Dual Assay Kit (Thermo Fischer Scientific) was used to determine luciferase activity. Each sample was normalized by dividing the activity of the test firefly luciferase by the expression of the control Renilla luciferase. Both tests were performed in triplicate.

### Wound healing assay

The migration of SKOV3 and ES-23 cells after transfection with plasmids, mimics, or inhibitors was assessed using a wound assay. The cells were inoculated in 6-well plates (1 × 106 cells), and the single cell layer was scratched with a 200 μL pipette tip. The cells were washed three times with PBS to remove cell fragments. RPMI-1640 without fetal bovine serum was added to each well and incubated at 37 °C and 5% CO_2_ for 48 h. Wound images were observed using a microscope at the same scratch location at 0, 24, and 48 h.

### Colony formation assay

For the colony formation assay, SKOV3 and ES-23 cells (5 × 10^2^) after transfection with plasmids, mimics, or inhibitors were seeded into 6-well plates. After 15-d routine culture, the generated colonies were fastened using 4% paraformaldehyde (Beyotime), stained with crystal violet (Beyotime), counted using ImageJ software (NIH, Bethesda, MD, USA), and photographed.

### RNA pull-down assays

Based on the manufacturer’s protocol (Thermo Fisher Scientific), the Pierce Magnetic RNA-Protein Pull-Down Kit was used for the RNA pull-down assay. Cell protein extracts were mixed with the specifically biotinylated RNA probes to circ_0000144, and magnetic beads with streptavidin were added for 1 h. The pull-down of the mixture was monitored by RT-qPCR.

### Western blotting

Radioimmunoprecipitation assay buffer was used to lyse the cells. An 8% SDS-PAGE gel was used to separate equal amounts of proteins, followed by transfer onto nitrocellulose membranes. The membranes were then blocked with 5% milk, followed by incubation with anti-ELK3 and anti-GAPDH antibodies (Santa, 1:1000). The membranes were then incubated with an HRP-conjugated secondary antibody. ECL reagents (Pierce, USA) were used to visualize proteins. The gray value of each band was analyzed using ImageJ (NIH, USA), with GAPDH as the endogenous reference.

### Target prediction

Circular RNA interactome (https://circinteractome.nia.nih.gov/index.html) and TargetScan (http://www.targetscan.org/mamm_31/) were used to identify the target genes of circ_0000144 and miR-610, respectively.

### Subcutaneous tumor model

Four-week-old female BALB/c nude mice were randomly divided into two groups (*n* = 6 per group). SKOV3 cells (5 × 106), which were transduced with circ_0000144 or NC vectors, were mixed in 150 μL of Matrigel. Then, the flanks of nude mice from different groups were handled by subcutaneous injection with a mixture. The equation for calculating the tumor volume was as follows: tumor volume = length × width × width/2. All animal experiments were performed in accordance with the relevant guidelines and regulations and were approved by First Hospital of Quanzhou Affiliated to Fujian Medical University.

### Statistical analysis

SPSS17.0 software was adopted for the experimental data analysis. Data are expressed as mean ± SD, and Student’s t-test was used for between-group comparisons. Differences between groups were analyzed using one-way analysis of variance. Survival analysis was conducted using Kaplan-Meier survival analysis. Differences were considered statistically significant at *p* < 0.05. All statistical analyses were performed using GraphPad Prism 5 software (GraphPad Software, La Jolla, CA, USA).

## Results

### Correlation of upregulated hsa_Circ_0000144 and Clinicopathological features in ovarian Cancer patients

**Fig. 1:**
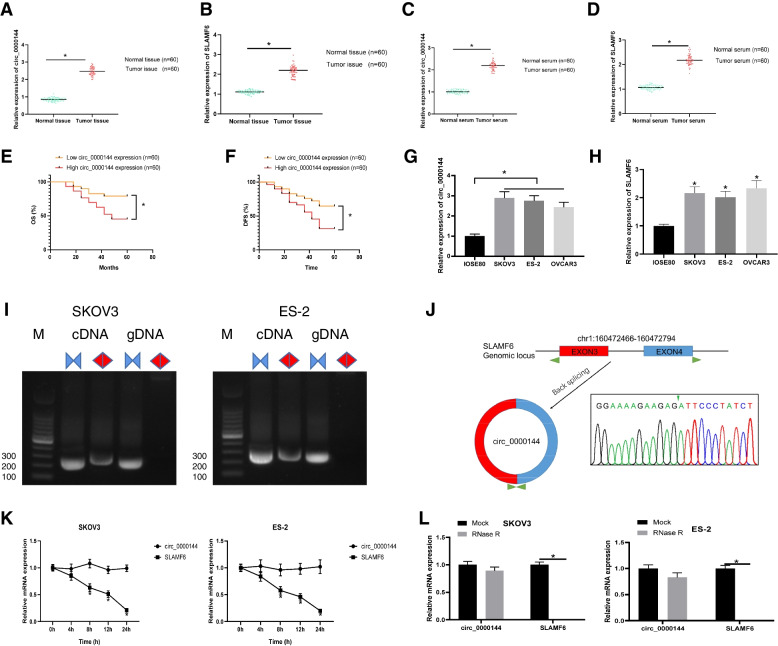
The expression of circ_0000144 was increased in ovarian cancer tissues, serum, and cells. The expression of circ_0000144 (**A**) and SLAMF6 (**B**) in 60 ovarian cancer tissues and paired adjacent normal tissues. The expression of circ_0000144 (**C**) and SLAMF6 (**D**) in 60 patient’s serum or 60 healthy controls was detected by qRT-PCR. Kaplan-Meier analysis showed that OS (**E**) rate and DFS (**F**) rate of patients with high circ_0000144 expression. The expression of circ_0000144 (**G**) and SLAMF6 (**H**) in cell lines was detected by qRT-PCR. (**I**) PCR assay with divergent and convergent primers were used for the amplification of cDNA and gDNA samples. In addition, PCR products of circ_0000144 were validated by Sanger sequencing (**J**). Actinomycin D treatment showed that circ_0000144 was more stable than linear SLAMF6 (**K**). In addition, RNase R digestion showed that linear mRNA SLAMF6 was digested while circ_0000144 was not (**L**). *P < 0.05

To determine the expression profile, we measured the expression of circ_0000144 in tissues, serum, and cells. The results showed that circ_0000144 expression was significantly higher in tumor tissues than in normal samples (Fig. 1A). We also detected expression of linear SLAMF6 mRNA, which is the linear isomer of circ_0000144. Expression of SLAMF6 was significantly higher in tumor tissues (Fig. 1B). In addition, circ_0000144 and SLAMF6 expression was also notably elevated in the serum of patients with ovarian cancer compared to that in normal samples (Fig. 1C and D). Kaplan-Meier analysis showed that the overall survival (OS) rate and disease-free survival (DFS) rates of patients with high circ_0000144 expression were significantly shorter than those with low circ_0000144 expression (Fig. 1E and F). In addition, we found circ_0000144 and SLAMF6 upregulation in ovarian cancer cell lines (SKOV3, ES-2, and OVCAR3) compared to that in normal human ovarian cells (IOSE80) (Fig. 1G and H). In addition, distinct PCR products with the expected size were amplified using convergent and divergent primers. The circ_0000144 were validated by PCR amplification using divergent primers from cDNA, but not from gDNA, of SKOV3 and ES-2 cell lines (Fig. 1I). The back-splicing sites were verified using Sanger sequencing (Fig. 1J). Moreover, the expression level of linear mRNA, rather than circ_0000144, was decreased in SKOV3 and ES-2 cells after RNase R digestion and actinomycin D treatment, suggesting a stable structure of hsa_circ_0000144 (Fig. 1K and L). Collectively, circ_0000144 was significantly upregulated in ovarian cancer tissues, serum, and cells, hinting that circ_0000144 affected ovarian cancer development.

### Circ_0000144 overexpression promotes ovarian Cancer progression in vitro and in vivo

**Fig. 2:**
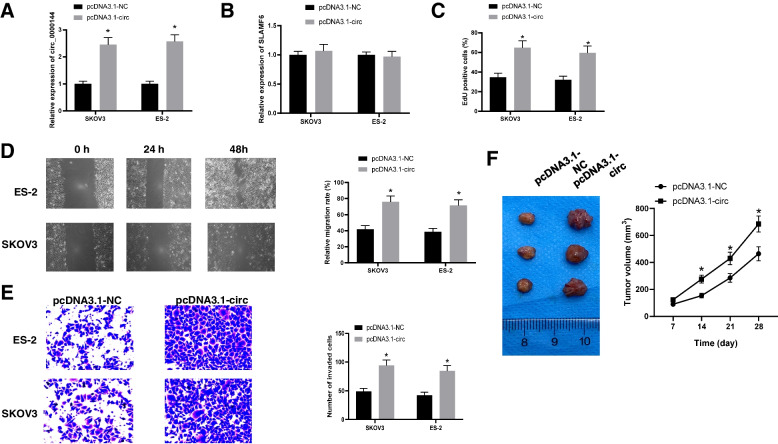
Circ_0000144 upregulation promoted ovarian cancer progression in vitro and in vivo. In SKOV3 and ES-2 cells transfected with pcDNA3.1-circ_0000144 or pcDNA3.1-NC, the expression of circ_0000144 (**A**) and SLAMF6 (**B**) was detected by qRT-PCR. **C** Schematic illustration of circ_0000144 from PCR products, and validation by Sanger sequencing. **D** Cell proliferation was detected by EDU assay. **E** and **F** Cell migration and invasion were monitored. **G** Tumor volume were measured in response to circ_0000144 overexpression. *P < 0.05

Circ_0000144 expression was the most upregulated in SKOV3 and ES-2 cells, as indicated in Fig. 1G; therefore, these two cell lines were selected for further study. To assess the biological function, circ_0000144 was overexpressed in the cells. qRT-PCR showed that circ_0000144 was upregulated in SKOV3 and ES-2 cells after transfection with circ_0000144 recombinant expression vector, but SLAMF6 showed no change in expression (Fig. 2A and B). To better investigate the function of circ_0000144, we performed an EdU assay to determine the function of circ_0000144 in SKOV3 and ES-2 cell proliferation. The results showed that circ_0000144 overexpression accelerated ovarian cancer cell proliferation (Fig. 2C). Migration and invasion capacities were also detected, and we found that circ_0000144 upregulation effectively increased the number of migrated and invaded SKOV3 and ES-2 cells (Fig. 2D and E). To test the biological role of circ_0000144 in vivo, nude mice were subcutaneously injected with SKOV3 cells transfected with pcDNA3.1-circ_0000144 or pcDNA3.1-NC. The results showed that the tumor volume in the circ_0000144 group was remarkably increased compared to that in the control group, indicating that circ_0000144 may promote ovarian cancer progression in vivo (Fig. 2F). Collectively, these findings indicate that circ_0000144 affects the occurrence and development of ovarian cancer, both in vitro and in vivo.

### Circ_0000144 directly bound to miR-610

**Fig. 3:**
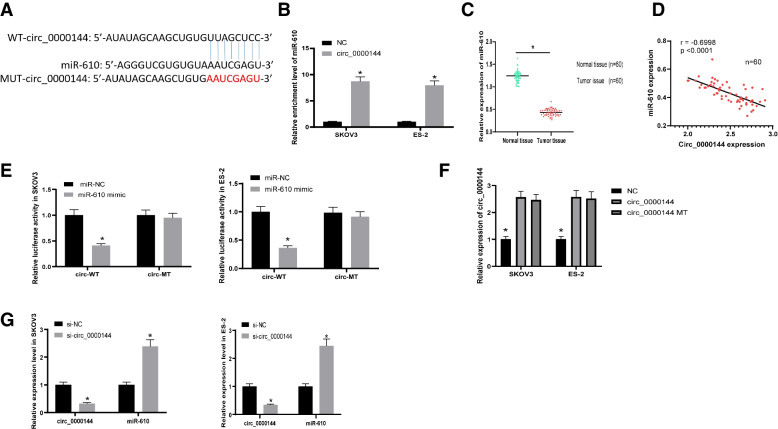
MiR-610 was a target of circ_0000144. **A** The binding site between circ_0000144 and miR-610 was analyzed by the bioinformatics tool circRNA interactome. **B** RNA pull-down assay examined the combination between circ_0000144 and miR-610. **C** Relative expression level of miR-610 was detected by qRT-PCR in ovarian cancer tissues. **D** Pearson’s correlation analysis for the correlation between circ_0000144 and miR-610 in ovarian cancer tissues. **E** The interplay between circ_0000144 and miR-610 was verified by luciferase reporter assay. **F** The circ_0000144 expression was detected after transfection of circ_0000144 or circ_0000144-mutation plasmid. **G** The expression of miR-610 in SKOV3 and ES-2 cells transfected with si-circ_0000144 or si-NC was checked by qRT-PCR. *P < 0.05

To explore the mechanisms by which circ_0000144 regulates ovarian cancer cell aggressive behavior, analysis of the bioinformatics tool circRNA interactome was carried out within SKOV3 and ES-2. Analysis of the circBank database revealed that circ_0000144 contains miR-610 binding sites (Fig. 3A). The results of the biotin-labeled RNA pull-down assay indicated that circ_0000144 could be pulled down by the biotin-miR-610 mimic rather than biotin-miRNA NC (Fig. 3B). In addition, qRT-PCR results showed that miR-610 was expressed at low levels in ovarian cancer tissues (Fig. 3C). Moreover, the expression level of circ_0000144 was significantly negatively correlated with miR-610 expression (Fig. 3D). A luciferase reporter assay was conducted using SKOV3 and ES-2 cells. Compared to miR-NC, the overexpression of miR-610 inhibited luciferase activity of wild-type circ_0000144 luciferase activity. After mutating the predicted binding site of circ_0000144, its inhibitory effect disappeared (Fig. 3E). In addition, transfection of circ_0000144 mutant plasmid did not affect the expression of circ_0000144, indicating binding sites mutation was not affect the circularization of circ_0000144 insert (Fig. 3). Subsequently, miR-610 expression increased after circ_0000144 knockdown in SKOV3 and ES-2 cells compared with si-NC (Fig. 3G). Taken together, these results indicated that miR-610 is a target of circ_0000144.

### Circ_0000144 regulates ovarian Cancer development through the sponge of miR-610

**Fig. 4:**
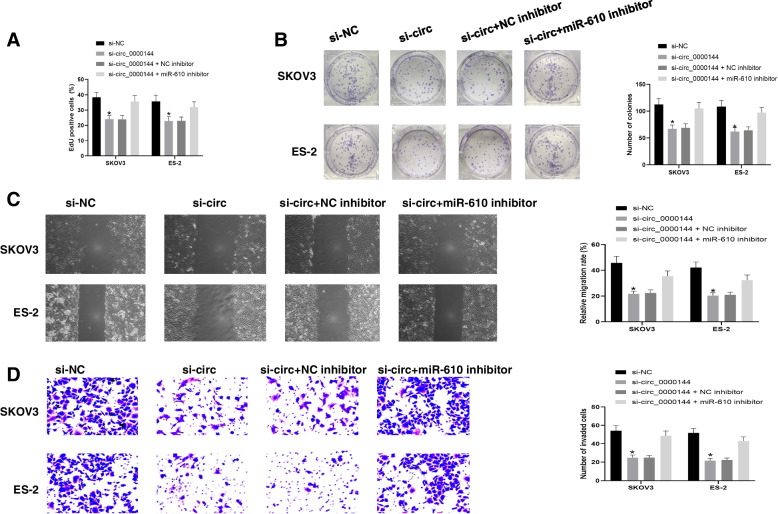
MiR-610 inhibition reversed the effects of circ_0000144 downregulation. In SKOV3 and ES-2 cells transfected with si-circ_0000144, si-NC, si-circ_0000144 + miR-610 inhibitor, or si-circ_0000144 + inhibitor NC, (**A**) cell proliferation was detected by EDU assay and (**B**) colony formation assay. **C** and **D** Cell migration and invasion were monitored. The results showed that si-circ_0000144 group was significantly different from si-NC or si-circ_0000144 + miR-610 inhibitor. *P < 0.05

Rescue experiments were performed in SKOV3 and ES-2 cells by transfection with si-circ_0000144 + NC inhibitor or si-circ_0000144 + miR-610 inhibitor, si-NC, or si-circ_0000144 serving as the respective controls. The EdU assay showed that knockdown of circ_0000144 slowed the growth of SKOV3 and ES-2 cells, while the miR-610 inhibitor partially increased cell growth (Fig. 4A). Consistently, circ_0000144 knockdown decreased the colony formation capacity of cells, while the miR-610 inhibitor partially increased cell colony formation (Fig. 4B). Furthermore, migration and invasion of SKOV3 and ES-2 cells were suppressed after circ_0000144 knockdown, and co-transfection with miR-610 inhibitor partially increased these abilities (Fig. 4C and D). Taken together, these results suggest that circ_0000144 regulates ovarian cancer progression by sponging miR-610.

### MiR-610 suppressed ovarian Cancer cell malignant development by binding to ELK3

**Fig. 5:**
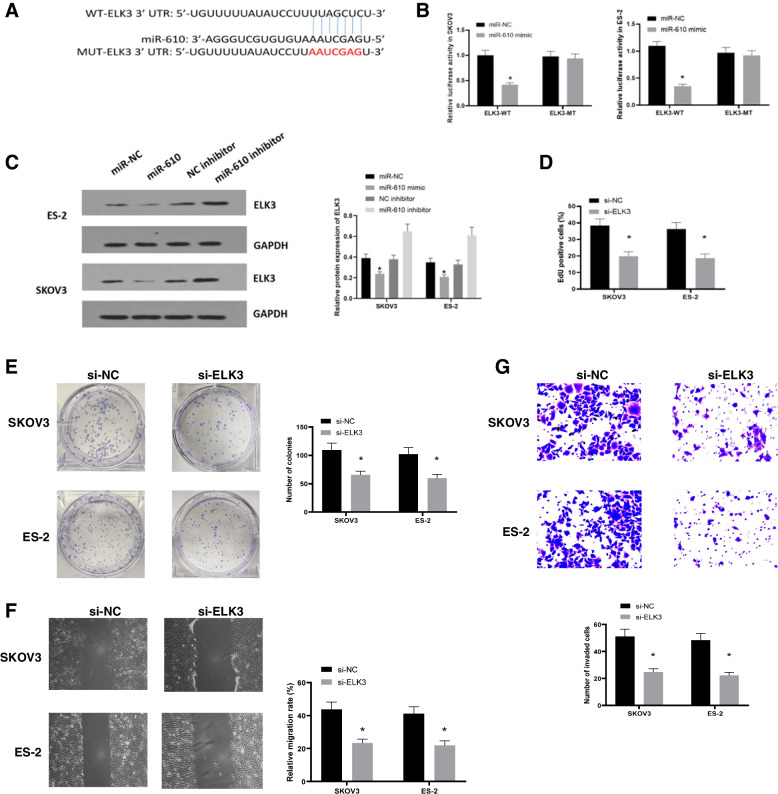
MiR-610 negatively regulated ELK3 in ovarian cancer cells. **A** The predicted binding sites between miR-610 and ELK3 5′UTR by TargetScan. **B** The luciferase reporter assays were performed to detect the correlation between. **C** The results showed that miR-610 overexpression group was significantly different from miR-610 inhibitor. MiR-610 negatively regulates ELK3 expression in SKOV3 and ES-2 cells. **D** In SKOV3 and ES-2 cells transfected with si-ELK3 or si-NC, (**D**) cell proliferation was detected by EDU assay and (**E**) colony formation assay. **F** and **G** Cell migration and invasion were monitored. *P < 0.05

The bioinformatics tool TargetScan predicted that there is an miR-610 binding site within the ELK3 non-coding region (Fig. 5A). Luciferase reporter assays were first performed in SKOV3 and ES-2 cells to determine the miR-610 function on ELK3 and verify the targeting relationship. miR-610 overexpression enhanced ELK3-WT-luciferase activity relative to miR-NC. Nonetheless, after the predicted binding site of ELK3 was mutated, its promotional effect disappeared (Fig. 5B). Subsequently, we found that miR-610 overexpression could inhibit ELK3 protein expression levels in SKOV3 and ES-2 cells, and ELK3 protein expression increased after incubation with the miR-610 inhibitor (Fig. 5C), indicating that miR-610 could act as an miRNA sponge that competes with miRNA for binding to ELK3. Functionally, we tested the ability of ELK3 to induce the proliferation of SKOV3 and ES-2 cells. The results showed that ELK3 knockdown reduced cell proliferation compared to that in the NC group (Fig. 5D and E). In line with this, we found that the knockdown of ELK3 suppressed the migration and invasion of cells (Fig. 5F and G). Taken together, these findings indicate that miR-610 impairs ELK3 expression to block ovarian cancer development in vitro.

### Circ_0000144 sponged miR-610 to regulate ELK3 expression

**Fig. 6:**
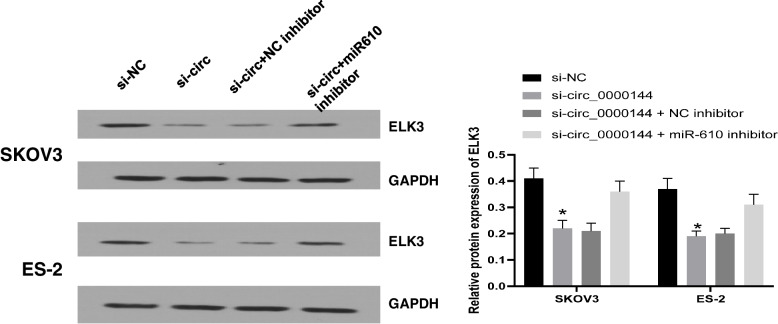
Circ_0000144 modulated ELK3 expression by targeting miR-610. SKOV3 and ES-2 cells were introduced with si-circ_0000144, si-NC, si-circ_0000144 + miR-610 inhibitor, or si-circ_0000144 + inhibitor NC, and the expression of ELK3 in these transfected cells was detected by Western blot. *P < 0.05

Expression analysis was performed in SKOV3 and ES-2 cells. As shown in Fig. 6, the expression of ELK3 impaired by transfection with si-circ_0000144 was partially strengthened by transfection with the si-circ_0000144 + miR-610 inhibitor, indicating that circ_0000144 downregulation could weaken the level of ELK3 by sponging miR-610.

## Discussion

Ovarian cancer, one of the most common cancers worldwide, remains an important human health problem that can lead to cancer-related threats. Thus far, the underlying regulatory mechanisms of ovarian cancer development remain unknown. Increasing evidence indicates that circRNAs contribute to elucidating the molecular mechanisms of cancer progression [21]. Circ_0000144 has been shown to participate in some types of cancer initiation and progression in previous studies [11, 13]. In the present study, we determined whether circ_0000144 enhances ovarian cancer tumorigenesis by regulating the downstream pathways.

Since circRNAs are involved in regulating ovarian cancer carcinogenesis, studies on their roles have received increased attention in recent years, including circCSPP1 [22], circular RNA Cdr1as [23], circEXOC6B, and circN4BP2L2 [24]. In a previous study, circ_0000144 was reported to be upregulated in gastric cancer cells and tissues [13]. In our study, we found that circ_0000144 expression was also increased in ovarian cancer cells and tissues. In addition, circ_0000144 also showed increased expression in peripheral blood samples of patients in our study and was correlated with the survival rate of patients, suggesting that circ_0000144 can be used as a diagnostic biomarker for ovarian cancer. The following functional experiments presented that circ_0000144 upregulation accelerated ovarian cancer cells viability, colony formation capacity, migration and invasion, and promoted tumorigenesis. Based on these results, we found that circ_0000144 plays a carcinogenic role in ovarian cancer, suggesting that circ_0000144 may be involved in the occurrence and development of ovarian cancer and is expected to become a therapeutic target of ovarian cancer, but the mechanisms still need to be studied.

It has been reported that miRNA sponge effects achieved by circRNA formation are now regarded as a general phenomenon in human malignancies [25]. Next, we analyzed the miRNAs known to be bound by circ_0000144 and identified miR-610 as a circ_0000144 associated miRNA. The sponge adsorption effect of circ_0000144 on miR-610 was further verified by dual-luciferase reporter gene and RNA pull-down assays. Growing evidence has shown that miR-610 dysregulation has been identified in different cancer types [20, 26, 27]. Nonetheless, the mechanisms by which miR-610 regulates the progression of ovarian cancer remain unknown. In this study, we found that miR-610 was downregulated in ovarian cancer tissues and negatively correlated with circ_0000144 in ovarian cancer tissues. In addition, low expression of miR-610 can promote proliferation, migration, and invasion of ovarian cancer cells. Further functional studies showed that miR-610 inhibitors functionally restored the ability of circ_0000144 knockdown in ovarian cancer cells. These results suggest that circ_0000144 regulates ovarian cancer progression by acting as an miR-610 sponge.

Together with miRNAs and their targets, the circRNA-miRNA-mRNA axis may function as an extensive regulatory network in gene expression, and their dysregulation may cause disease progression, including cancer development [28]. In the present study, we found that ELK3 was co-overexpressed with circ_0000144 in ovarian cancer cells. ELK3 has been reported to participate in cancer genesis and development, including gastric [29], breast [17], and liver cancers [30]. Although previous studies have also demonstrated that ELK3 expression is altered in ovarian cancer cell lines and tumors through overexpression of miR-378 [19], the regulatory mechanism by which ELK3 regulates ovarian cancer remains unclear. According to our findings, ELK3 was predicted to be a direct target of miR-610 by bioinformatics analysis. Moreover, knockdown of ELK3 inhibited proliferation, migration, and invasion of ovarian cancer cells. This phenomenon is similar to that observed for circ_0000144 in ovarian cancer cells. We further demonstrated that miR-610 suppressed ELK3 expression and that circ_0000144 could promote ELK3 expression by sponging miR-610 in ovarian cancer cells, which enhanced ovarian cancer cell proliferation, migration, and invasion. Therefore, we speculate that circ_0000144 acts as a sponge for miR-610 to enhance ELK3 expression and inhibit ovarian cancer cells. Therefore, the circ_0000144/miR-610/ELK3 network may promote a new treatment strategy for patients with ovarian cancer.

Although our study demonstrated the relationship between circ_0000144 and clinicopathological features of ovarian cancer, the present work still has some limitations, including the lack of a deeper understanding of the underlying molecular mechanism between miR-610 and circ_0000144, and functional experiments were only performed in the cell line. Therefore, further studies in nude mice are required to confirm our conclusions.

## Conclusions

This study provides the first evidence that circ_0000144 is significantly upregulated in ovarian cancer cells, tissues, and serum samples. Meanwhile, circ_0000144 overexpression promotes ELK3 protein expression through the sponging of miR-610, causing ovarian cancer cell proliferation, migration, and invasion. In addition, circ_0000144 overexpression markedly accelerated ovarian cancer tumorigenesis in a mouse xenograft model. These data indicate that circ_0000144 is a novel candidate therapeutic biomarker for ovarian cancer and a specific clinical diagnostic and prognostic biomarker and therapeutic target for ovarian cancer.

## Data Availability

The datasets used and/or analysed during the current study are available from. the corresponding author upon reasonable request.
